# CP-QRRT*: A Path Planning Algorithm for Hyper-Redundant Manipulators Considering Joint Angle Constraints

**DOI:** 10.3390/s25051490

**Published:** 2025-02-28

**Authors:** Tianya Wang, Guoliang Ma, Lisong Xu, Rui Yu

**Affiliations:** School of Energy and Power Engineering, Nanjing University of Science and Technology, Nanjing 210094, China; skyedge03@njust.edu.cn (T.W.);

**Keywords:** hyper-redundant manipulators, path planning, joint angle constraints, RRT, PSO

## Abstract

A novel algorithm (CP-QRRT*) is proposed for the path planning tasks of hyper-redundant manipulators (HRMs) in confined spaces, addressing the issues of unmet joint angle constraints, redundant planning paths, and long planning times present in previous algorithms. First, the PSO algorithm is introduced to optimize the random sampling process of the RRT series algorithms, enhancing the directionality of the random tree expansion. Subsequently, the method of backtracking ancestor nodes from the Quick-RRT* algorithm is combined to avoid getting trapped in local optima. Finally, a constraint module designed based on the maximum joint angle constraints of the HRM is implemented to limit the path deflection angles. Simulation experiments demonstrate that the proposed algorithm can satisfy the joint angle constraints of the HRM, and the planned paths are shorter and require less time.

## 1. Introduction

Due to the continuous advancement of science and technology, the structures of devices such as aircraft engines and nuclear power plants have become increasingly complex, making it difficult for traditional manipulators to access them [1]. Snake-like manipulators, inspired by the flexible and resilient characteristics of snakes in nature, have become powerful tools for operations in confined spaces and hazardous environments, thanks to their compact structure, flexible movement, and ability to operate in narrow spaces [2]. The technology related to snake-like manipulators designed for unstructured environments has become one of the key research directions in robotics [3,4]. Among these, hyper-redundant manipulators (HRM) with a spinal structure are a common configuration [5].

The HRM features a slender arm and redundant degrees of freedom, enabling it to perform complex tasks in confined spaces [6]. As a result, it is widely used in fields such as aerospace, facility maintenance, and healthcare [7]. However, since HRMs often operate in narrow, constrained environments filled with obstacles, traditional inverse kinematics [8] methods (such as pseudo-inverse solutions based on the Jacobian matrix) are no longer applicable [9]. Consequently, ensuring that the HRM avoids collisions with the surrounding environment during operation becomes a challenge. However, the Follow-The-Leader [10] (FTL) strategy addresses this challenge by designating the HRM’s end effector as the "leader" with the remaining segments following its movement [11]. This effectively transforms the problem into a path planning issue in space. It is sufficient to plan the path of the end effector within the confined workspace and then have the HRM follow that path, thereby ensuring collision-free operation for the entire system. Compared to traditional methods, this approach offers significant advantages when navigating narrow spaces [12].

Common path planning algorithms include A* algorithm [13], Dijkstra’s algorithm [14], Artificial Potential Field algorithm [15] and Model-based algorithm [16]. However, these algorithms have certain limitations and suboptimal performance when applied to path planning in confined workspaces: The A* algorithm and Dijkstra algorithm require traversing of the map structure, resulting in poor performance in high-dimensional spaces. The Artificial Potential Field algorithm suffers from high computational complexity in complex environments and is prone to falling into local optima. Meanwhile, the Model-based algorithm exhibits limited scenario adaptability, necessitating manual adjustment of modal functions. In contrast, the Rapidly-exploring Random Tree (RRT) algorithm, which explores complex environments through random sampling and expansion, can quickly navigate such spaces [17]. Additionally, RRT performs exceptionally well in high-dimensional state spaces, providing an effective solution for path planning in complex environments [18].

In recent years, numerous scholars have conducted research on RRT-based algorithms. In 2000, Kuffner and LaValle introduced vertex sampling optimization [19]. In 2003, Urmson and Simmons presented the sampling heuristic RRT algorithm [20]. In 2016, Li et al. proposed stable sparse RRT (S-RRT) based on a drain function to remove unnecessary nodes, thus improving planning speed. In 2020, Liu, Feng proposed a step-size optimization strategy [21] to improve the performance of the RRT algorithm. In 2019, Chao, Liu, and others integrated the RRT* algorithm with the D*Lite algorithm, resulting in the DL-RRT* algorithm [22], which continuously refines the existing path by sampling the search graph obtained during grid search, thus improving path planning efficiency in highly dynamic environments. In 2021, Wang, Li, and collaborators introduced a rapid bidirectional path planning algorithm, the Bidirectional Rapidly-exploring Random Tree (KB-RRT*) [23], which effectively prunes the random tree. In 2023, Ji et al. proposed ERRT* algorithm [24], which replaces traditional RRT* line segment connections with ellipsoid-shaped connections between nodes. And the ellipsoid dimensions are designed based on manipulator arm dimensions. In 2024, Ganesan and colleagues proposed a hybrid sampling-based path planning method, termed Hybrid-RRT*, which generates samples using both non-uniform and uniform samplers, effectively addressing the slow convergence issue of uniform sampling and the exploration limitations of non-uniform sampling [25].

Although significant progress has been made in research on RRT-based algorithms, several limitations remain when applying them to path planning for HRMs. These include the inability to satisfy joint angle constraints, path redundancy, and long planning time. To address these issues, this paper proposes a novel Constraint-PSO-QRRT* algorithm (CP-QRRT*), which builds upon the ancestor node backtracking method originally proposed by Quick-RRT*. The algorithm innovatively integrates the Particle Swarm Optimization (PSO) algorithm [26] to optimize the random sampling process and designs a Constraint module to impose a limit on the maximum deflection angle of the path. Finally, the effectiveness of the proposed algorithm is demonstrated through simulation comparison experiments conducted in a confined environment.

## 2. Preliminaries

**Figure 1:**
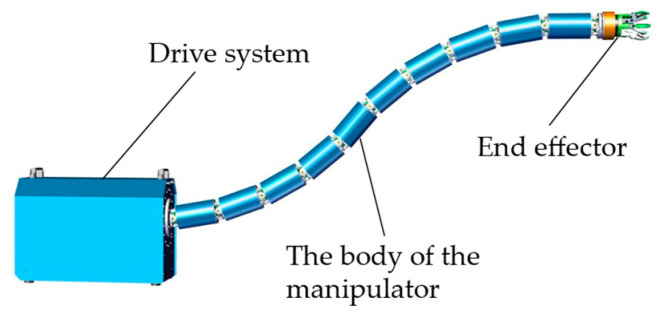
Structural design of the HRM.

The overall structural design of the HRM is shown in Figure 1, which consists of the drive system, the manipulator body, and the end effector. The drive system includes a multi-channel, ring-arranged motor control system, communication equipment, and edge computing devices. The body of the HRM is composed of multiple vertebral segments, with each pair of adjacent segments connected by universal joints. The deflection of these segments is achieved through the retraction and extension of ropes. The end effector, in this case, is a gripper, but it can be replaced with task-specific equipment according to operational requirements.

### 2.1. Structural Characteristics of HRMs

**Figure 2:**
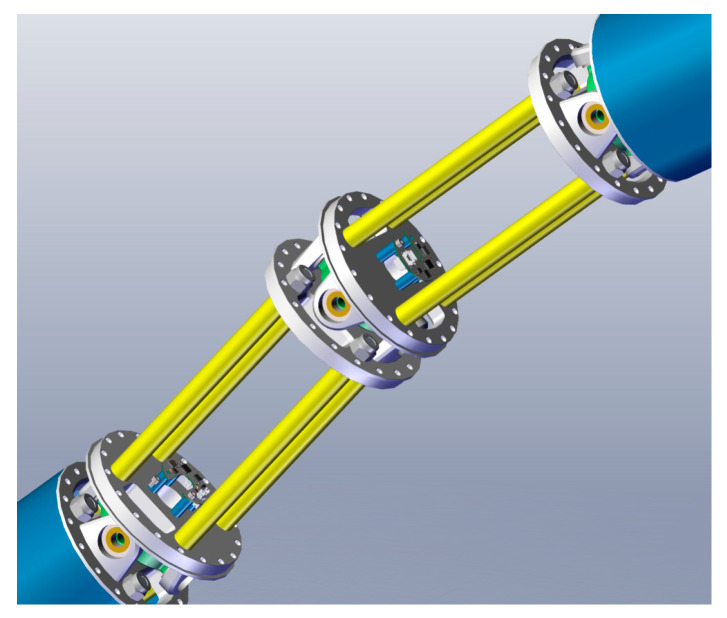
The details of the connection between two segments.

The details of the connection between two adjacent vertebral segments are shown in Figure 2. Due to the structural characteristics, when the joint angle between the two segments exceeds a certain threshold, the adjacent segments are constrained from further rotation due to interference, thereby imposing a maximum angular constraint on the joint.

### 2.2. Geometric Analysis of Path Limitations Induced by Joint Angle Constraints

**Figure 3:**
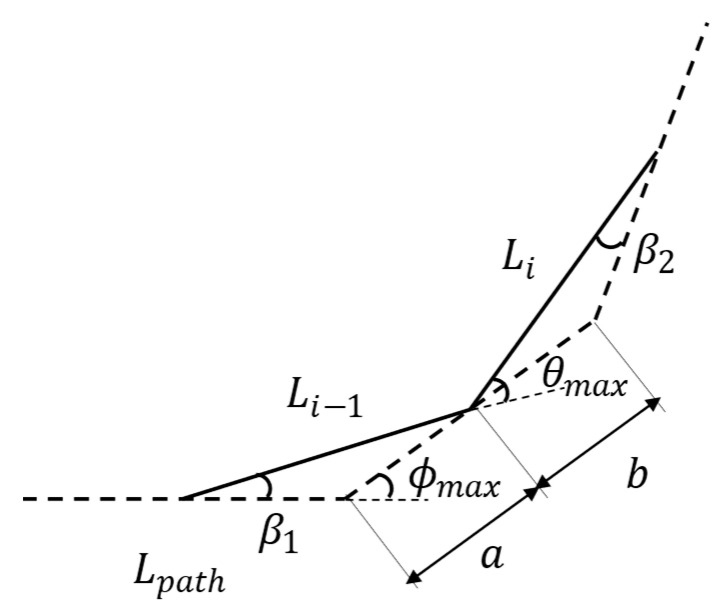
Geometrical schematic of the movement of the HRM along the path.

Due to the adoption of the Follow-the-Leader strategy, once the path of the end effector is planned, the HRM must track this path to complete the motion planning task. During path tracking, the HRM can be equivalently treated as a polyline. Consequently, given the maximum angular constraints of the joints, the planned path must also adhere to specific angular limitations. The geometric relationship between the maximum joint angle and the maximum path deflection angle is illustrated in Figure 3, where the solid line represents the HRM, the dashed line represents the path, $\theta_{max}$ is the maximum joint angle, and $\phi_{max}$ is the maximum path deflection angle.

When the step length of the path and the length of the HRM’s segments are equal, i.e., $L_{path}=L_{i-1}=L_{i}$, the geometric relationship can be expressed as follows:

(1) $$\left\{\;\;\begin{array}{c} \theta_{max}=2\phi_{max}-\beta_{2}-\beta_{1}\\ \frac{a}{\sin\beta_{1}}=\frac{L_{i-1}}{\sin\phi_{max}}\\ \frac{b}{\sin\beta_{2}}=\frac{L_{i}}{\sin\phi_{max}}\\ a+b=L_{path}\\ L_{path}=L_{i-1}=L_{i} \end{array}\right.$$

The simplified relationship between the maximum joint angle and the maximum path deflection angle is given by:

(2) $$\theta_{max}=2\left[\phi_{max}-arcsin\left(\frac{\sin\phi_{max}}{2}\right)\right]$$

When the step length of the path is shorter than the length of the HRM’s segment, i.e., $L_{path}={kL}_{i-1}={kL}_{i}$, where *k* ∈ (0, 1), there are two cases that will cause the HRM to reach the maximum joint angle constraint: the first case is when the end of the segment moves to the endpoint of the path (i.e., a = 0 or b = 0), and the second case is when the end of the segment moves to the midpoint of the path (i.e., a = b). The geometric relationship can be expressed as follows, according to the research by Jia et al. [27]:

(3) $$\theta_{\mathrm{end}\;}=\left(2N_{\mathrm{end}\;}+1\right)\phi_{max}-2arcsin\left[ksin\left(\frac{N_{\mathrm{end}\;}+1}{2}\phi_{max}\right)sin\left(\frac{N_{\mathrm{end}\;}\phi_{max}}{2}\right)/sin\left(\frac{\phi_{max}}{2}\right)\right]$$

(4) $$\theta_{\mathrm{mid}\;}=2N_{mid}\phi_{max}-2arcsin\left[k{sin}^{2}\left(\frac{N_{mid}\phi_{max}}{2}\right)/tan\left(\frac{\phi_{max}}{2}\right)\right]$$

where $N_{\mathrm{end}\;}$ indicates the number of turning points contained between the two ends of the HRM in the first case above; $N_{mid}$ indicates the number of turning points contained between the two ends of the HRM in the second case.

The greater of the two angles is considered the maximum joint angle, i.e., $\theta_{max}=max\left(\theta_{\mathrm{end}\;},\theta_{\mathrm{mid}}\right)$. Given the maximum joint angle $\theta_{max}$, the maximum deflection angle of the path can be obtained under different path steps (i.e., different *k* values), as shown in Table 1:

Through theoretical analysis, when the path step length is equal to the length of the manipulator segment, the maximum path deflection angle of the planned path should be less than $\phi_{max}$. In this case, the maximum joint angle of the HRM will not exceed $\theta_{max}$, resulting in a feasible path for the HRM.

For the HRM, in order to perform operations in confined spaces, the Follow-the-Leader strategy is employed, with the end effector as the leader and the first n-1 segments of the manipulator as followers. This transforms the problem into a path planning issue for a point within the confined space, which simplifies the motion planning process and divides it into two components: end effector path planning and path tracking. It is only necessary to plan the path of the end effector within the confined workspace, and by implementing path tracking control for the manipulator, it ensures that the manipulator as a whole avoids collisions during operation. This paper focuses exclusively on the path planning aspect, aiming to plan a feasible path for subsequent path tracking, and does not delve into the details of path tracking methods.

## 3. Related Works

### 3.1. RRT

The RRT algorithm is a widely used path planning method in fields such as robotics and unmanned aerial vehicles (UAVs) [28]. Its main idea is to construct a tree that spans the entire space by randomly sampling points in the environment, with the goal of finding a path from the start point to the target.

**Figure 4:**
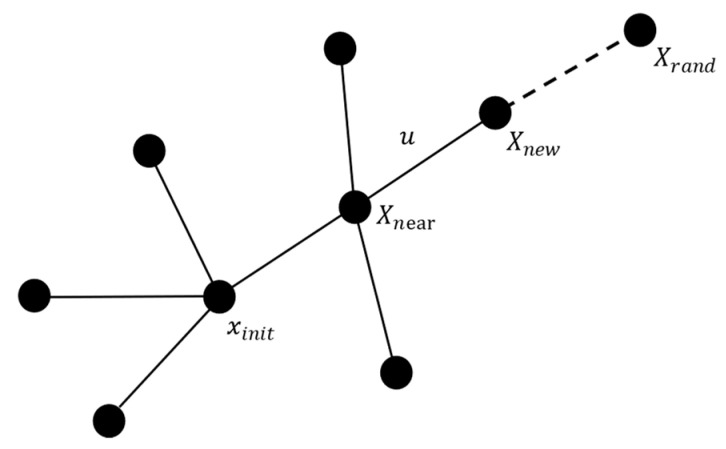
Schematic diagram of RRT algorithm.

As shown in Figure 4, the steps of the RRT algorithm are as follows:

1. Randomly sample a point $X_{rand}$ in the free space.
2. Find the node $X_{near}$ in the random tree T that is closest to $X_{rand}$.
3. Calculate the distance between $X_{near}$ and $X_{rand}$. If the distance is greater than the step size *u*, move a distance *u* from $X_{near}$ towards $X_{rand}$ to obtain a new node $X_{new}$; otherwise, generate the new node $X_{new}$ directly at the position of $X_{rand}$.
4. If there is a straight-line path between $X_{new}$ and $X_{near}$, add $X_{new}$ to the random tree *T*, with $X_{near}$ as its parent; otherwise, proceed to the next iteration.

The input to the RRT algorithm includes the initial point, the goal point, and the map, while the output is a graph containing a feasible path. The classic RRT algorithm performs random sampling throughout the entire space, providing good robustness, particularly in high-dimensional spaces. However, the path generated by the classic RRT is generally suboptimal, and it lacks a subsequent optimization process. As a result, it faces issues such as low vertex utilization, slow convergence, and path instability.

### 3.2. RRT*

The RRT* algorithm proposed by Karaman et al. [29] is an extension of the RRT algorithm that introduces two additional procedures:

1. *Parent Search*: This procedure identifies a suitable low-cost parent node for the candidate node.
2. *Rewire*: This procedure reconnects existing nodes to the candidate node to improve the overall path quality.

The RRT* algorithm is an improvement over the RRT algorithm. Unlike the classic RRT, which focuses solely on connecting the start and goal points, RRT* also considers the reconnection of neighboring nodes during the expansion of new nodes to minimize path costs. RRT* is asymptotically optimal, meaning that, with a sufficiently large number of samples, it can converge onto the optimal solution.

**Figure 5:**
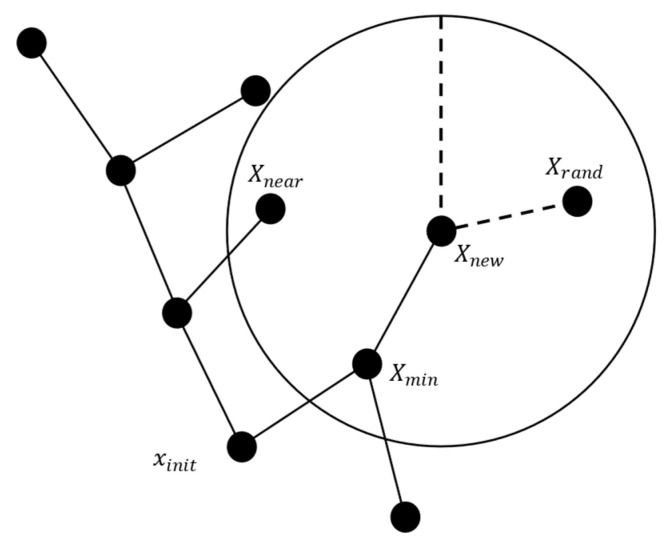
Schematic diagram of the rewiring process of RRT* algorithm.

The RRT* algorithm includes a rewiring procedure (*Rewire*) when selecting the parent node. Specifically, within a neighborhood centered at $X_{new}$ with radius *r*, the algorithm identifies the node that, when connected to $X_{new}$, results in the minimum path cost (i.e., the length of the path from the start to $X_{new}$). This node $\;X_{min}$ is then selected as the new parent node for $X_{new}$, replacing the previous nearest node $X_{near}$. A diagram illustrating the rewiring process is shown in Figure 5.

### 3.3. Quick-RRT*

The Quick-RRT* (QRRT*) algorithm, proposed by Jeong et al. [30], combines RRT* with an additional search procedure that examines the ancestors of the selected parent node. Compared to the paths generated by RRT*, this algorithm utilizes the triangle inequality property to expand the set of potential parent nodes, thereby producing paths with lower costs [31].

**Figure 6:**
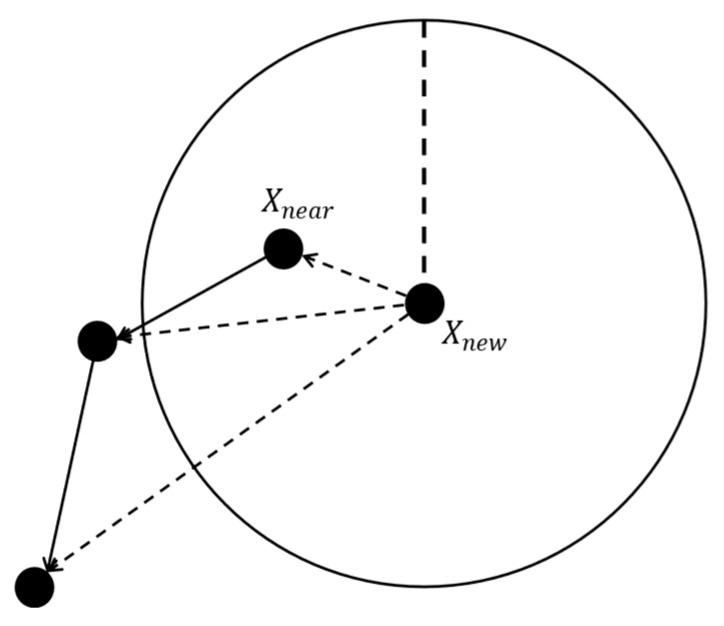
Schematic diagram of backtracking ancestor node process of QRRT* algorithm (*Depth* = 2).

When the state $X_{new}$ is added to the tree, QRRT* searches the set of nearby vertices $X_{near}$ to find the vertex that, like in RRT*, offers the lowest-cost path to $X_{new}$. However, it also considers the ancestors of $V_{near}$ based on the depth parameter: *Depth*, as illustrated in Figure 6 (where *Depth* is set to 2). If the triangle inequality holds for the cost function, and as long as the path is collision-free, the parent node of any vertex $X_{near}\;$∈ $V_{near}$ will always generate a lower-cost path than $X_{near}$.

**Figure 7:**
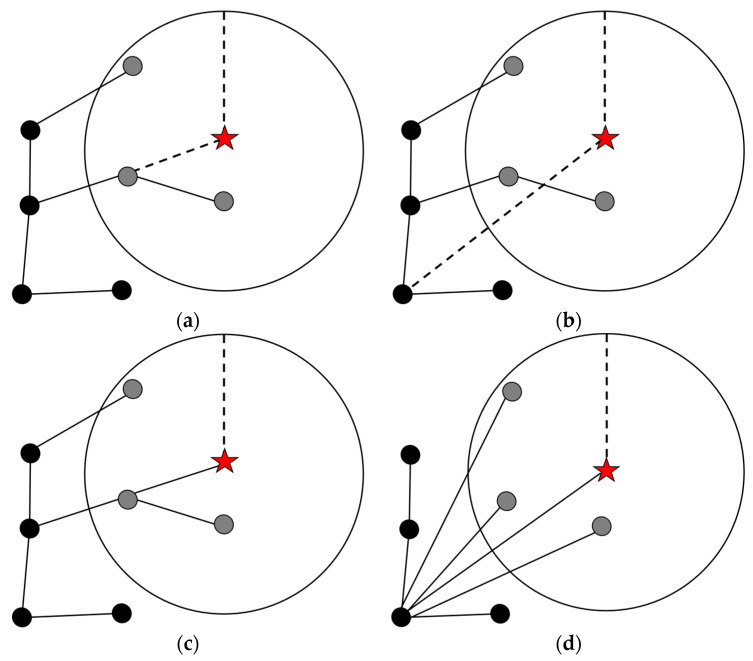
Schematic diagram of RRT*, QRRT* algorithms. (**a**) *ChooseParent* procedure of RRT* algorithm; (**b**) *ChooseParent* procedure of QRRT* algorithm; (**c**) *Rewire* procedure of RRT* algorithm; (**d**) *Rewire* procedure of QRRT* algorithm.

Figure 6 illustrates the working principle of QRRT* (*Depth* = 2), compared to RRT*. When a new sample $X_{new}$ (the red star in Figure 7) is added, RRT* only searches the nearby vertices $X_{near}$ (the gray points) and connects $X_{new}$ to one of them. However, QRRT* searches both $X_{near}$ and the ancestors of $X_{near}$, as shown in Figure 7b. As a result, QRRT* finds a better path compared to RRT*. Figure 7c,d show the significant differences in the *Rewire* procedure between RRT* and QRRT*, where QRRT* not only straightens the path to $X_{new}$ but also straightens the paths to the nearby vertices.

## 4. Proposed Algorithm

### 4.1. PSO-QRRT* Algorithm

The basic principles of the existing RRT series algorithms have been introduced earlier. In these algorithms, the tree is expanded by performing random sampling in the feasible space, and the expansion of the random tree lacks directionality. Therefore, this paper proposes a sampling procedure to improve the random sampling process and optimize the expansion of the path random tree. The proposed method uses the PSO algorithm [26] to optimize the sampling process, utilizing PSO-optimized sampling points to expand the tree rather than relying entirely on random sampling, thus making the expansion of the random tree more directional. However, through theoretical analysis, it is not difficult to find that since only the next sampling point is considered, this method is prone to getting stuck in local optima, whereas the QRRT* algorithm considers ancestor nodes. By combining the QRRT* algorithm with the PSO-optimized random sampling method and using QRRT*’s ancestor search procedure to select better parent nodes for candidate nodes, this issue can be avoided. The pseudocode for the PSO-QRRT* algorithm is shown in Algorithm 1.
**Algorithm 1:** PSO-QRRT***INPUT**: Environment ***map***; Initial point $x_{init}$; Goal $x_{goal};$ Maximum number of samples ***k***; Threshold ***tol***; Step size δ; *Depth **d***; Neighborhood radius ***r***.
**OUTPUT**: Random tree T. 1.$V\leftarrow x_{init};E\leftarrow \emptyset ;T\leftarrow \left(V,E\right)$
2. **for** i = 1 **to** k **do**
3.  $x_{rand}\leftarrow GetSample\left(obj\right)$
4.  $x_{near}\leftarrow Nearest\left(V,x_{rand}\right)$
5.  $x_{new}\leftarrow NewVertex\left(x_{near},x_{rand},\delta \right)$
6.  $\sigma \leftarrow Steer\left(x_{near},x_{new}\right)$
7.  $if\;\mathrm{CollisionFree}\left(\sigma ,map\right)\;then$
8.    $X_{neighbor}\leftarrow Near\left(x_{new},V,r\right)$ 9.    $X_{ancestry}\leftarrow Ancestry\left(X_{neightbor},V,d\right)$ 10.   $x_{p}\leftarrow \mathrm{ChooseParent}\left(x_{new},x_{near},X_{neighbor}\cup X_{ancestry},\sigma \right)$ 11.   $\sigma_{p}\leftarrow \mathrm{ChooseParent}\left(x_{new},x_{near},X_{neighbor}\cup X_{ancestry},\sigma \right)$ 12.   $T=\left(V,E\right)\leftarrow Re\mathrm{connect}\left(x_{new},x_{p},\sigma_{p},T=\left(\begin{array}{c} V,E \end{array}\right)\right)$ 13.   $T\leftarrow Rewire\left(x_{new}\cup X_{ancestry},X_{neighbor}\right)$ 14.   $if\;Distance\left(x_{new},x_{goal}\right)<tol\;\;\;then$ 15.     **return**
T 16.   **end if** 17.  **end if** 18.**end for** 19.**return**
T


To optimize the random sampling process, the algorithm proposed in this paper designs the *GetSample* function, which utilizes the PSO algorithm to optimize the sampling points. The pseudocode for the *GetSample* function is shown in Algorithm 2.
**Algorithm 2:** 
$GetSample\left(obj\right)$
**INPUT**: Population size ***s***; Inertia weight ***w***; Number of iterations ***n***; Damping coefficient ***wDamping***; Learning coefficient $c_{1}$, $c_{2}$; Fitness function ***fitness()***.
**OUTPUT:** Random state ***randState***.
1.*samplelist* ← *SampleInit (s)*2. **Initialize** *pos, vel, pBest, gBest* 3.  **for** i = 1 **to** n **do** 4.        *vel*
←
*vUpdate*(*pos, vel, pBest, gBest, w, c1, c2*) 5.        *pos*
←
*pos + vel*6.        *cost*
←
*cUpdate*(*pos,*
$x_{goal}$*, fitness*()) 7.        *pBest*
←
*pBUpdate*(*pos, cost, pBest*) 8.        *gBest*
←
*gBUpdate*(*pos, cost, gBest*) 9.        *w*
←
w * wDamping 10.  **end for** 11. *randState* ← *gBest.Pos* 12. **return** *randState*

In this function, firstly, particles are randomly initialized in the sampling space, with their positions representing potential sampling points. Subsequently, a user-defined fitness function, referred to as ‘*fitness*’, is utilized to evaluate both *pbest* and *gbest*. Finally, iterative updates are conducted, and the optimized sampling points are generated at the end.

The update rules for the velocity and position of the particles are described by the following equations:

(5) $${vel}_{i}^{new}=w\cdot {vel}_{i}^{old}+c_{1}\cdot r_{1}\cdot \left(pbest_{i}-{pos}_{i}\right)+c_{2}\cdot r_{2}\cdot \left(gbest-{pos}_{i}\right)$$

(6) $${pos}_{i}^{new}={pos}_{i}^{old}+{vel}_{i}^{new}$$

where ${vel}_{i}$ is the velocity of the particle, ${pos}_{i}$ is the position of the particle, w is the inertia weight, $c_{1}$ and $c_{2}$ are the learning coefficient, $r_{1}$ and $r_{2}$ are random numbers, $pbest_{i}$ is the individual best position of the particle, and gbest is the global best position.

**Figure 8:**
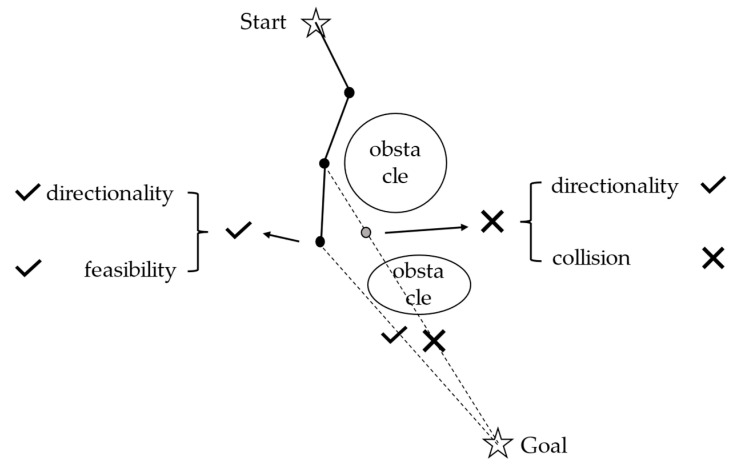
Schematic diagram of the fitness function operation process.

The fitness function should comprehensively consider the following two factors to balance directionality and feasibility, as illustrated in Figure 8:

1. The distance to the goal.
2. Collision penalty: This involves checking whether the line connecting the current particle to the goal intersects with any obstacles. If a collision occurs, it indicates that extending in this direction will inevitably lead to a collision with an obstacle. Therefore, a penalty value should be imposed to encourage the particle to deviate from this point.

Consequently, the fitness function can be represented as follows:

(7) $$Fitness=\omega_{1}\cdot \mathrm{DistanceToGoal}+\omega_{2}\cdot \mathrm{CollisionPenalty}$$

(8) $$\mathrm{CollisionPenalty}=\left\{\begin{array}{c} P\;\;\;\;if\;collision\;occurs\\ 0\;\;\;\;if\;no\;collision\;\;\;\;\;\;\; \end{array}\right.$$

where $\omega_{1}$ and $\omega_{2}$ are weights that adjust the relative importance of *DistanceToGoal* and *CollisionPenalty* in the fitness function.

### 4.2. Constraint Module

Based on the method of ancestor node backtracking of the QRRT* algorithm and utilizing the PSO algorithm to optimize the random sampling process, the PSO-QRRT* algorithm was proposed. However, as previously discussed, for HRMs, due to the existence of joint angle constraints, the path planner should satisfy certain deflection angle constraints to ensure that the path is feasible for the HRMs. To meet this constraint, this paper proposes a maximum deflection angle constraint module (Constraint module) to improve the PSO-QRRT* algorithm, thereby proposing a Constraint-PSO-QRRT* algorithm (CP-QRRT*). The pseudocode for the CP-QRRT* algorithm is shown in Algorithm 3.
**Algorithm 3:** CP-QRRT***INPUT**: Environment ***map***; Initial point $x_{init}$; Goal $x_{goal};$ Maximum number of samples ***k***; Threshold ***tol***; Step size δ; *Depth*
***d***; Neighborhood radius ***r***.
**OUTPUT**: Random tree T.
1.$V\leftarrow x_{init};E\leftarrow \emptyset ;T\leftarrow \left(V,E\right)$
2. **for** i = 1 **to** k **do**
3.  $x_{rand}\leftarrow GetSample\left(obj\right)$
4.  $x_{near}\leftarrow Nearest\left(V,x_{rand}\right)$
5. $x_{new}\leftarrow NewVertex\left(x_{near},x_{rand},\delta \right)$
6.  $\sigma \leftarrow Steer\left(x_{near},x_{new}\right)$
7.  $if\;\mathrm{CollisionFree}\left(\sigma ,map\right)\;then$
8.   $x_{new}\leftarrow Constraint\left(x_{rand},x_{near},T\right)$
9.   $\sigma \leftarrow Steer\left(x_{near},x_{new}\right)$
10.   $if\;\mathrm{CollisionFree}\left(\sigma ,map\right)\;then$
11.    $X_{neighbor}\leftarrow Near\left(x_{new},V,r\right)$
12.    $X_{ancestry}\leftarrow Ancestry\left(X_{neightbor},V,d\right)$
13.   $x_{p}\leftarrow \mathrm{ChooseParent}\left(x_{new},x_{near},X_{neighbor}\cup X_{ancestry},\sigma \right)$
14.   $\sigma_{p}\leftarrow \mathrm{ChooseParent}\left(x_{new},x_{near},X_{neighbor}\cup X_{ancestry},\sigma \right)$
15.   $T=\left(V,E\right)\leftarrow Re\mathrm{connect}\left(x_{new},x_{p},\sigma_{p},T=\left(\begin{array}{c} V,E \end{array}\right)\right)$
16.   $T\leftarrow Rewire\left(x_{new}\cup X_{ancestry},X_{neighbor}\right)$
17.    $if\;\;\;\;Distance\left(x_{new},x_{goal}\right)<tol\;\;\;then$
18.     return T
19.    **end if**
20.   **end if**
21.  **end if**
22.**end for**
23.**return**
T


**Figure 9:**
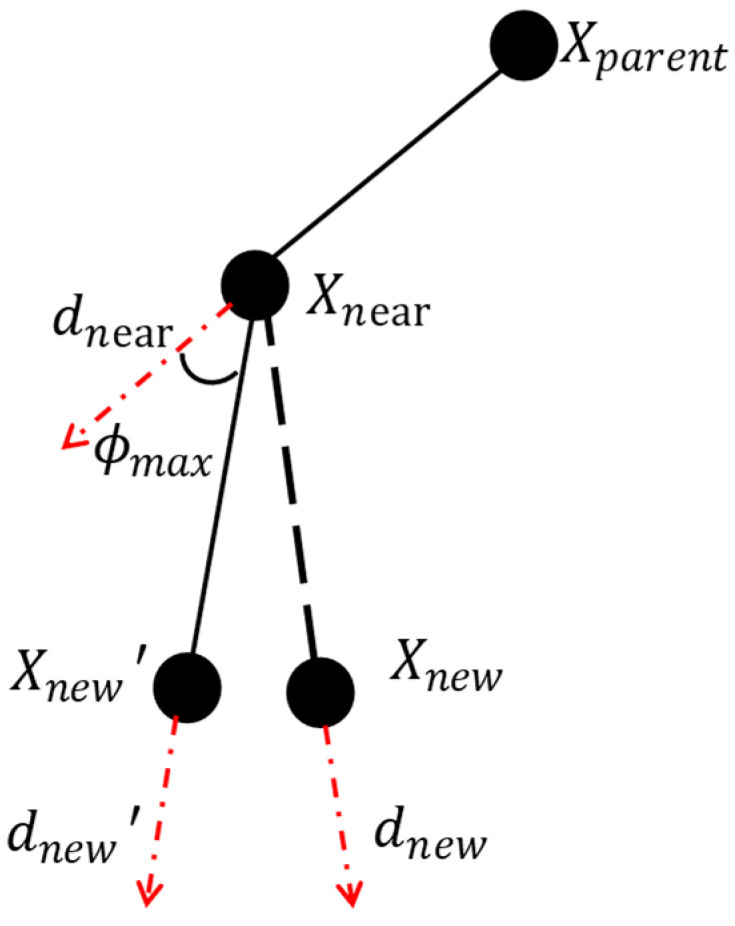
Schematic diagram of expanding $d_{new}$.

To limit the maximum deflection angle of the path, the Constraint module should include both the calculation of the deflection angle and the decision criteria. The principle is as follows:

1. First, calculate the direction vector $d_{new}$ of the line connecting $x_{new}$ and $x_{near}$, as well as the direction vector $d_{near}$ of the line connecting $x_{near}$ and its parent node.
2. Calculate whether the angle between the two vectors satisfies the maximum path deviation angle constraint. If it does not, replan the direction using the *Reset* function (i.e., deflect $d_{near}$ by $\phi_{max}$ to obtain a new direction vector $d_{new}^{’}$). Finally, return the expansion direction of the new node $x_{new}$. This process is illustrated in Figure 9.

The pseudocode for the Constraint module is shown in Algorithm 4:
**Algorithm 4:** 
$Constraint\left(x_{rand},x_{near},T\right)$
1. $d_{new}=x_{new}-x_{near}$
2. $d_{near}=T\left(near,parent\right)$
3. $if\;arccos\left(\frac{d_{rand}\cdot d_{near}}{\left|d_{rand}\right|\cdot \left|d_{rand}\right|}\right)>\phi_{max}$
4.    $d_{new}=Reset\left(d_{near},d_{new},\phi_{max}\right)$
5. **end if** 6. **return** $d_{new}$


## 5. Simulations and Experiment

### 5.1. Environment

**Figure 10:**
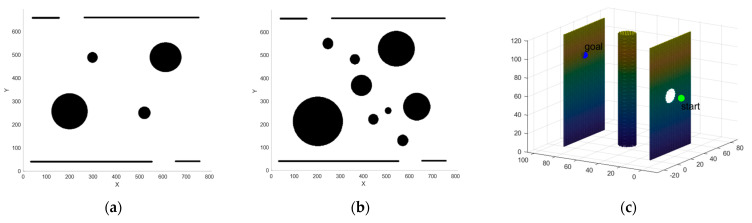
Different maps used for simulations. (**a**) Map1; (**b**) Map2; (**c**) Map3.

HRMs are capable of operating in confined spaces, accessing environments where human intervention is challenging, and navigating around obstacles to accurately reach the desired position, making them well-suited for tasks in narrow and hazardous areas. To validate the effectiveness of the proposed algorithm, this section constructs two 2D obstacle maps and one 3D map that simulate confined spaces, as shown in Figure 10. The 2D obstacle maps measure 800 × 650 and feature two walls (top and bottom), as well as some black circular obstacles of varying sizes to simulate obstacles found in real-world operational scenarios. Meanwhile, the 3D obstacle map measures 100 × 100 × 120 and features two walls, as well as a cylinder to simulate obstacles found in real-world operational scenarios. The start and goal positions of the 2D maps are (200, 650) and (600, 40), respectively, and the start and goal positions of the 3D map are (30, 0, 60) and (30, 90, 60), respectively. The simulation experiments presented in this paper were conducted on a Windows 11 operating system equipped with an Intel Core i9-8950HK processor (clock speed: 2.90 GHz) and 16 GB of RAM. 

### 5.2. Simulation 1

**Figure 11:**
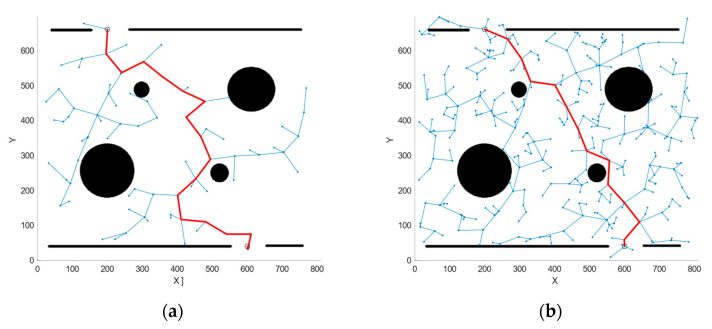

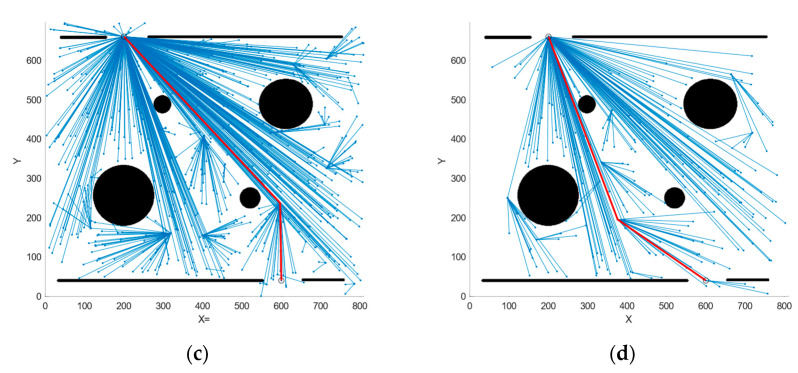
Simulation results of each algorithm for Map1. (**a**) RRT algorithm; (**b**) RRT* algorithm; (**c**) *Q*RRT* algorithm; (**d**) PSO-QRRT* algorithm.

**Figure 12:**
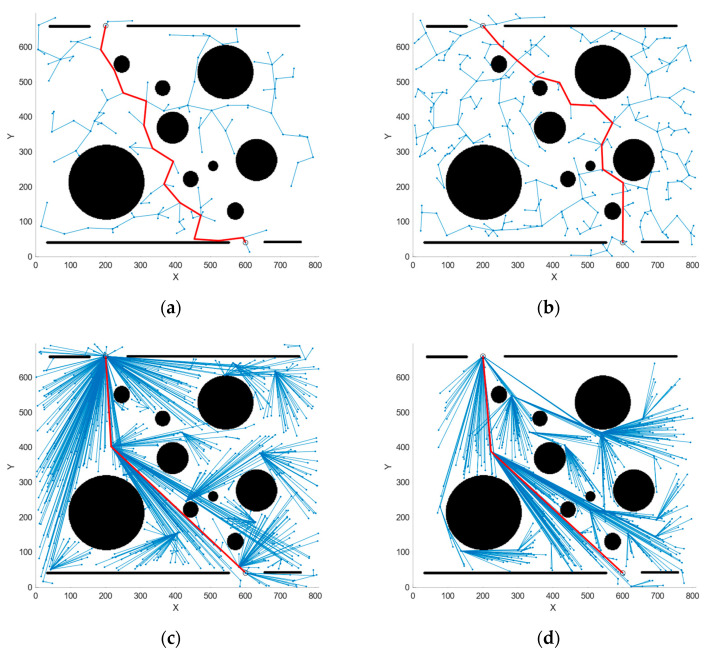
Simulation results of each algorithm for Map2. (**a**) RRT algorithm; (**b**) RRT* algorithm; (**c**) *Q*RRT* algorithm; (**d**) PSO-QRRT* algorithm.

**Figure 13:**
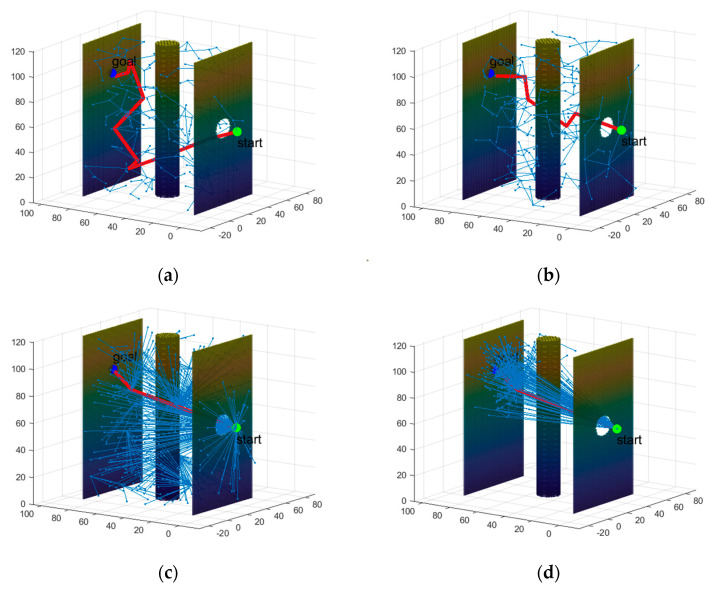
Simulation results of each algorithm for Map3. (**a**) RRT algorithm; (**b**) RRT* algorithm; (**c**) *Q*RRT* algorithm; (**d**) PSO-QRRT* algorithm.

First, path planning was conducted using the RRT, RRT*, QRRT*, and PSO-QRRT* algorithms on the obstacle maps. The parameter *Depth* for both the QRRT* and PSO-QRRT* algorithms was set to 2, while the population size for the PSO-QRRT* algorithm was configured to 20. The paths obtained by each algorithm are shown in Figure 11, Figure 12 and Figure 13, where the red line denotes the final planned path, and the blue lines represent the random trees generated during the planning process. A comparison of the performance metrics of each algorithm is provided in Table 2.

The simulation results above indicate that, within the obstacle maps, both the RRT and RRT* algorithms exhibit relatively short planning times; however, the paths they generate are longer and exhibit redundancy. The QRRT* algorithm significantly enhances the quality of the planned path, resulting in a shorter path; however, it requires a longer planning time. In contrast, the PSO-QRRT* algorithm proposed in this paper effectively balances path length with planning speed. It produces the shortest path among the four algorithms while also demonstrating shorter planning times compared to QRRT*.

As mentioned earlier, there is a certain relationship between the maximum joint angle and the maximum path deflection angle. When the path step size is equal to the length of the manipulator segment, the calculation of the maximum path deflection angle is as follows: Given that the maximum joint angle $\theta_{max}$ is 35°, substituting $\theta_{max}=\frac{\pi }{6}$ into Equation (2) yields a maximum path deflection angle $\phi_{max}$ of approximately 33.53°.

However, as shown in Table 2, the maximum deflection angles of the paths planned by all four algorithms exceed the limit of 33.53°, rendering them infeasible for the HRM. Therefore, it is necessary to incorporate a maximum deflection angle constraint into the path planning algorithm to ensure that the maximum deflection angle of the final planned path does not exceed 33.53°. Consequently, improvements should be made to the existing path planning algorithms by introducing the maximum deflection angle constraint module (Constraint module) previously mentioned.

### 5.3. Simulation 2

Based on the simulation results and Table 2, it can be observed that the paths generated by RRT and RRT* algorithms exhibit considerable redundancy, making the introduction of the Constraint module into these algorithms impractical. Therefore, the Constraint module is introduced into the QRRT* and PSO-QRRT* algorithms for simulation, allowing for a comparison of the performance of the C-QRRT* and CP-QRRT* algorithms.

**Figure 14:**
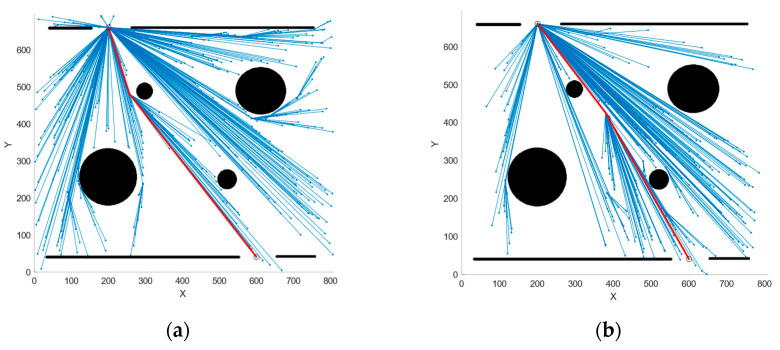
Simulation results of each algorithm considering maximum deflection angle constraints for Map1. (**a**) C-QRRT algorithm; (**b**) CP-QRRT* algorithm.

**Figure 15:**
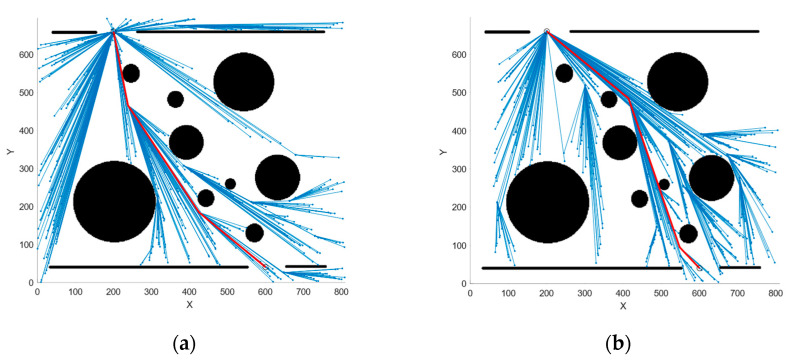
Simulation results of each algorithm considering maximum deflection angle constraints for Map2. (**a**) C-QRRT algorithm; (**b**) CP-QRRT* algorithm.

**Figure 16:**
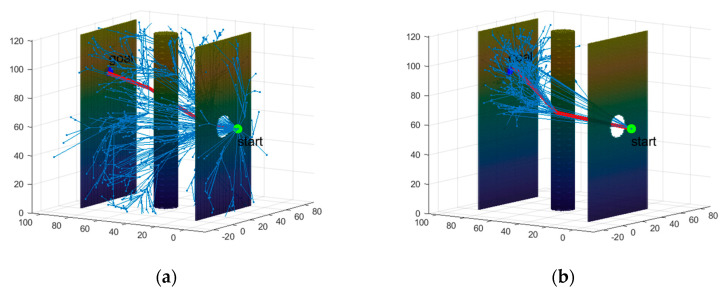
Simulation results of each algorithm considering maximum deflection angle constraints for Map3. (**a**) C-QRRT algorithm; (**b**) CP-QRRT* algorithm.

Simulations were conducted in the same environment, and the final planned path along with the random tree is illustrated in Figure 14, Figure 15 and Figure 16. A comparison of the performance metrics of the two algorithms is provided in Table 3.

The simulation results indicate that after the introduction of the Constraint module, the maximum deflection angle of the paths planned by the two algorithms does not exceed 33.53°, rendering them feasible for the HRM. This result demonstrates the effectiveness of the proposed Constraint module in keeping the joint angles within the allowable range and highlights its potential for broader applications.

### 5.4. Experiment

**Figure 17:**
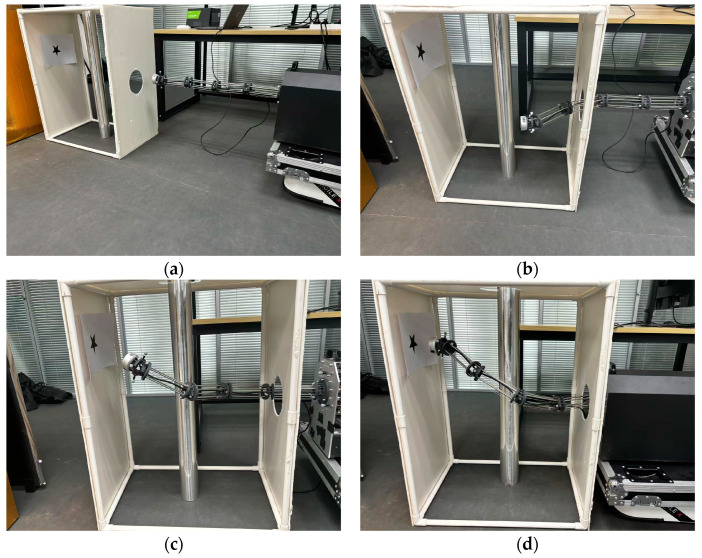
Experimental results at different times. (**a**) *t* = 0 s; (**b**) *t* = 15 s; (**c**) *t* = 30 s; (**d**) *t* = 45 s.

The experiment in this section is designed to verify that the path planned by the proposed algorithm is practically trackable for HRMs. The experimental scenario is consistent with the 3D map constructed in the simulation part of the paper. In practical applications, it is necessary to ensure that the end-effector reaches the target position with a specific orientation, and given the inherent errors in path tracking, the actual motion trajectory of the HRM may exhibit minor deviations from the planned path. However, these deviations do not affect the validation of the path’s feasibility. As stated at the end of Section 2, this paper focuses exclusively on the path planning aspect, aiming to generate a feasible path for subsequent path tracking, and does not explore the specifics of path tracking methods. The experimental results at different times are shown in Figure 17, which demonstrate that the path planned by the proposed algorithm in this paper is practically trackable for the HRM.

## 6. Discussion

**Figure 18:**
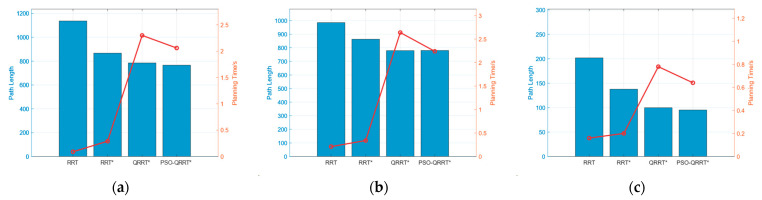
Results of Simulation1. (**a**) Map1; (**b**) Map2; (**c**) Map3.

The comparison of planning time and path length of the four algorithms is presented in Figure 18. Simulation 1 demonstrates that both RRT and RRT* exhibit path redundancy, whereas QRRT* is capable of generating shorter paths but requires a longer planning time. However, the PSO-QRRT* algorithm proposed in this paper effectively balances path length with planning speed. It produces the shortest path among the four algorithms while also demonstrating shorter planning times compared to QRRT*. 

However, as shown in Table 2, the maximum deflection angles of the paths planned by all four algorithms exceed the limit of 33.53°, rendering them infeasible for the HRM. Therefore, this paper introduces the previously mentioned Constraint module and compares the performance of C-QRRT* and CP-QRRT* algorithms in Simulation 2.

**Figure 19:**
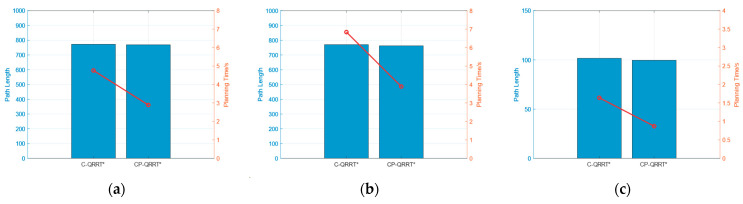
Results of Simulation2. (**a**) Map1; (**b**) Map2; (**c**) Map3.

The comparison of planning time and path length between the C-QRRT* and CP-QRRT* algorithms is presented in Figure 19. After introducing the maximum deflection angle constraint, the planning time of CP-QRRT* is only 60.71% of that of C-QRRT* in Map1, 56.87% in Map2, and 53.05% in Map3, significantly reducing the overall planning time. The path lengths obtained by both algorithms are nearly identical, with no path redundancy observed. Therefore, based on the simulation results and analysis above, it can be concluded that the CP-QRRT* algorithm proposed in this paper not only meets the joint angle constraints but also achieves shorter planning times and path lengths.

## 7. Conclusions

When HRMs operate in confined spaces, RRT-based algorithms often encounter challenges such as path redundancy, lengthy planning times, and difficulties in satisfying joint angle constraints. This paper proposes two key improvements: integrating the PSO algorithm to optimize the random sampling process of RRT-based algorithms and designing a module (Constraint module) to limit the maximum deflection angle of the path. Through these enhancements, the Constraint-PSO-QRRT* algorithm (CP-QRRT*) is ultimately proposed.

The simulation analysis indicates that, through the integration of the PSO algorithm to optimize the random sampling process, the proposed CP-QRRT* algorithm generates a more directional expansion tree, leading to shorter paths without redundancy. Moreover, due to the enhanced directionality of the expansion tree, the frequency of the *Rewire* procedure is reduced compared to QRRT*, resulting in shorter planning times. Furthermore, the re-planning step introduced by the Constraint module makes the planning speed advantage of CP-QRRT* over C-QRRT* even more apparent.

Consequently, the CP-QRRT* algorithm proposed in this paper is capable of planning paths that adhere to the joint angle constraints of the HRM, with reduced planning times and shorter path lengths, thereby demonstrating its potential for broader applications.

Additionally, in our future work, we intend to explore and investigate more state-of-the-art algorithms. By comprehensively analyzing the strengths and limitations of these algorithms, we aim to develop a novel path planning algorithm with enhanced performance metrics. This endeavor will involve a rigorous evaluation of state-of-the-art algorithms, with the objective of synthesizing their advantageous features while mitigating their respective shortcomings, ultimately contributing to the advancement of the field.

## Figures and Tables

**Table 1:** Maximum deflection angle of the path under different *k* values ($\theta_{max}=35{}^{\circ}$).

k	0.1	0.3	0.5	0.7	1.0
$N_{\mathrm{end}\;}$	10	3	2	1	0
$N_{mid}$	10	3	2	1	1
$\phi_{max}$	3.44°	10.13°	17.11°	21.64°	33.53°

**Table 2:** Comparison of the performance metrics of each algorithm. (The data of path length and planning time in each group represent the average of 10 experimental trials).

Map	Performance Metrics	RRT	RRT*	QRRT*	PSO-QRRT*
Map1	Path Length	1136.81	867.21	785.06	765.72
	Planning Time/s	0.09	0.29	2.30	2.06
	Maximum Path Deflection Angle/°	127.68	86.91	53.77	37.82
Map2	Path Length	984.68	862.83	778.61	780.03
	Planning Time/s	0.21	0.34	2.64	2.24
	Maximum Path Deflection Angle/°	118.60	88.57	44.07	42.39
Map3	Path Length	201.72	137.64	99.69	95.07
	Planning Time/s	0.16	0.20	0.78	0.64
	Maximum Path Deflection Angle/°	130.73	112.68	57.91	43.70

**Table 3:** Comparison of the performance metrics of the two algorithms. (The data of path length and planning time in each group represent the average of 10 experimental trials).

Map	Performance Metrics	C-QRRT*	CP-QRRT*
Map1	Path Length	772.60	768.94
	Planning Time/s	4.76	2.89
	Maximum Path Deflection Angle/°	30.55	33.53
Map2	Path Length	770.32	762.03
	Planning Time/s	6.84	3.89
	Maximum Path Deflection Angle/°	28.76	31.64
Map3	Path Length	101.62	99.63
	Planning Time/s	1.64	0.87
	Maximum Path Deflection Angle/°	33.53	33.48

## Data Availability

The original datasets of this study are outlined within the article. For further inquiries, please contact the corresponding author.

## Abbreviations

The following abbreviations are used in this manuscript:

HRM	Hyper-redundant manipulator
FTL	Follow-The-Leader
RRT	Rapidly-exploring Random Tree
QRRT*	Quick-RRT*
P-QRRT*	PSO-Quick-RRT*
CP-QRRT*	Constraint-PSO-Quick-RRT*
